# Gastric Neuroendocrine Tumor: A Report of a Rare Case

**DOI:** 10.7759/cureus.103901

**Published:** 2026-02-19

**Authors:** Emmanuel Mduma, Ndigwake E Mallango, Swafaa Z Abdallah, Andrew K Jackson, Elijah Ussiri

**Affiliations:** 1 Clinical Oncology, Rabininsia Memorial Hospital, Dar es Salaam, TZA; 2 Radiology, Rabininsia Memorial Hospital, Dar es Salaam, TZA; 3 Anatomical Pathology, Muhimbili University of Health and Allied Sciences, Dar es Salaam, TZA; 4 Pathology, Rabininsia Memorial Hospital, Dar es Salaam, TZA; 5 Surgery, Rabininsia Memorial Hospital, Dar es Salaam, TZA

**Keywords:** case report, chromogranin a, gastric neuroendocrine tumor, gnet, partial gastrectomy

## Abstract

Gastric neuroendocrine tumors (gNETs) are rare gastric neoplasms that often present with non-specific symptoms and may mimic other submucosal gastric lesions. We report a case of a WHO Grade 1 gastric neuroendocrine tumor in a 67-year-old male who presented with a 7-month history of epigastric pain, early satiety, and melena. Esophagogastroduodenoscopy revealed a broad-based ulcerated fundal mass, initially suspected to be a gastrointestinal stromal tumor. Histopathological examination with immunohistochemistry confirmed a well-differentiated neuroendocrine tumor with strong chromogranin A positivity. Contrast-enhanced computed tomography demonstrated a localized submucosal fundal lesion without nodal or distant metastasis. The patient underwent partial gastrectomy with complete excision of the tumor. Histology confirmed clear resection margins. The postoperative course was uneventful. This case emphasizes the diagnostic importance of histopathology and immunohistochemistry and supports surgical resection as effective treatment for localized, low-grade gastric neuroendocrine tumors.

## Introduction

Gastric neuroendocrine tumors (gNETs) are uncommon epithelial neoplasms arising from enterochromaffin-like cells of the gastric mucosa and represent a small fraction of all gastric malignancies [1]. They comprise approximately 0.1-0.6% of all gastric cancers, with an estimated incidence of 2-3 cases per 100,000 persons per year [2]. Although generally indolent, larger lesions may present with gastrointestinal bleeding, abdominal pain, anemia, or obstructive symptoms [3]. Endoscopic and radiological findings may resemble other submucosal gastric tumors, particularly gastrointestinal stromal tumors (GISTs), making histopathological confirmation essential [4]. We report a rare case of a WHO Grade 1 gastric neuroendocrine tumor initially suspected to be a GIST. To the best of our knowledge, this is the first reported case of gNET in our setting and the first documented case in Tanzania.

## Case presentation

**Figure 1:**
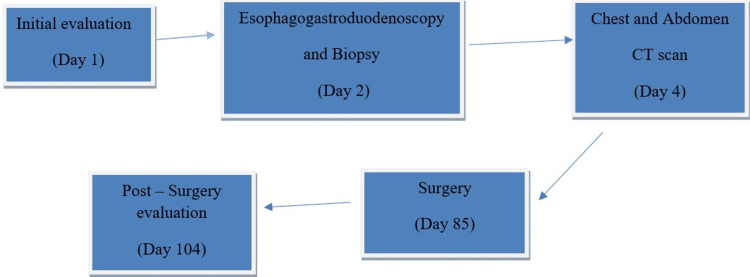
Timeline of important clinical events

A 67-year-old male of African ethnicity, a known hypertensive on regular amlodipine 10 mg once daily and losartan/hydrochlorothiazide 50 mg/12.5 mg once daily, presented to our facility (Day 1; Figure 1) with a 7-month history of epigastric pain. The pain was burning in nature and associated with early satiety and passage of black stools. There was no history of vomiting, significant weight loss, or prior gastrointestinal surgery.

**Figure 2:**
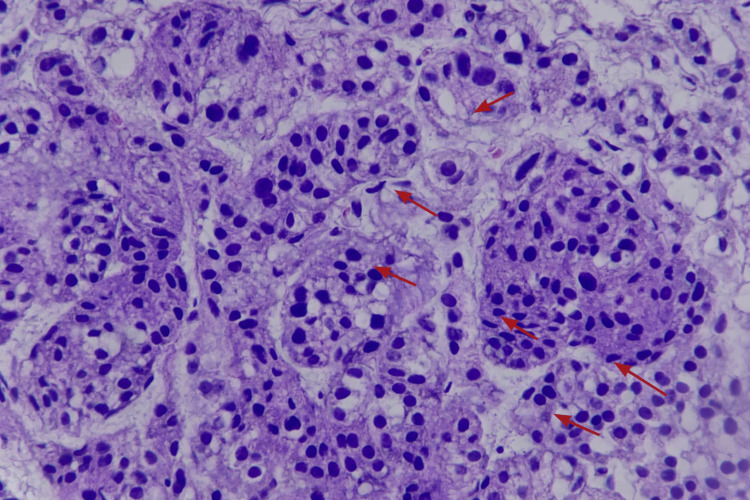
Histopathological features of the gastric neuroendocrine tumor (hematoxylin and eosin stain, X400) Hematoxylin and eosin-stained sections demonstrate a well-differentiated neuroendocrine tumor composed of uniform epithelioid cells arranged in compact nests and trabeculae separated by delicate fibrous septa. The tumor cells exhibit moderate eosinophilic to amphophilic cytoplasm with well-defined cell borders. The nuclei are round to ovoid with smooth contours, finely stippled (“salt-and-pepper”) chromatin, and inconspicuous nucleoli. Mitotic activity was low (<2 mitoses per 10 high-power fields), consistent with low-grade (WHO Grade 1) classification.

**Figure 3:**
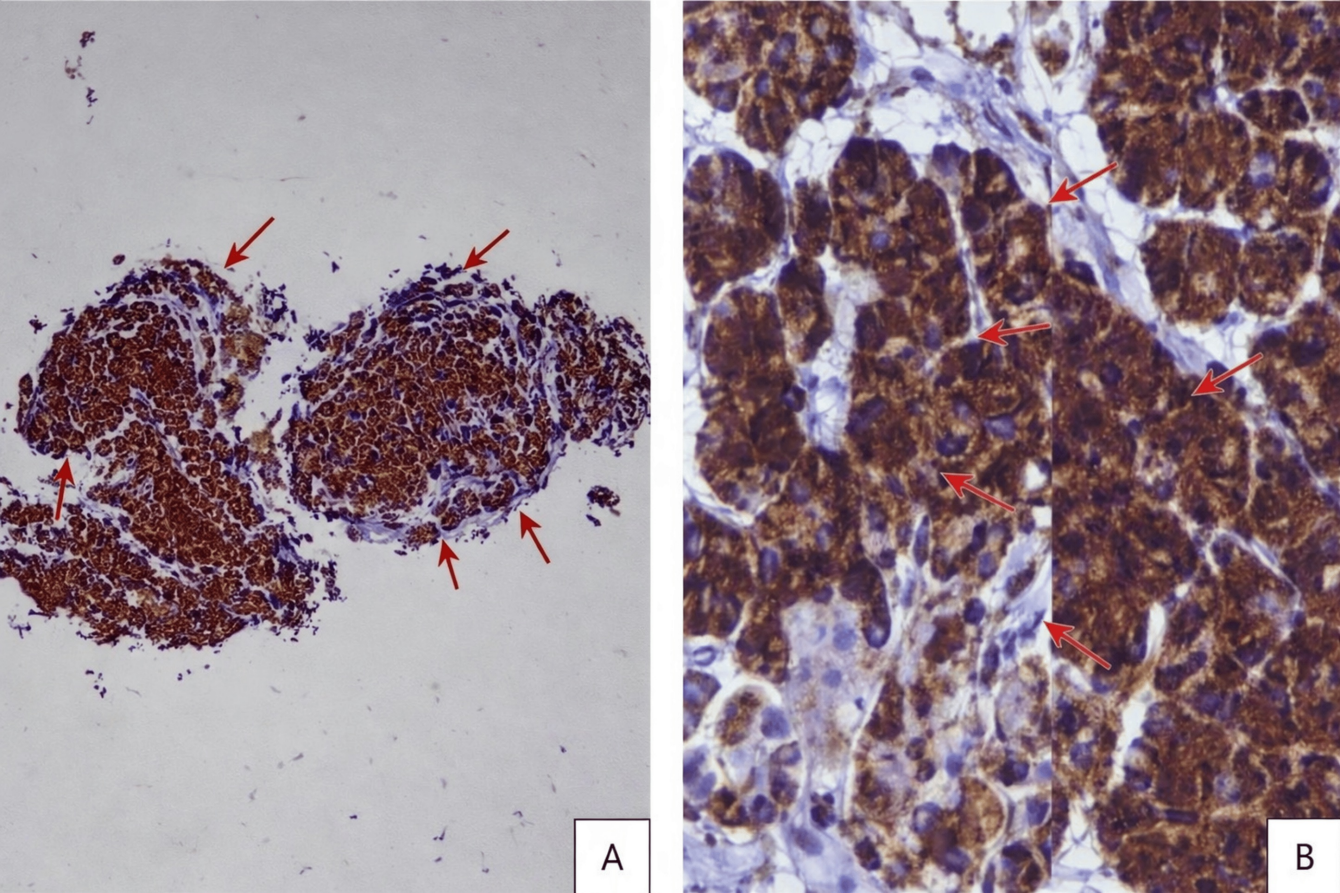
Immunohistochemistry demonstrating diffuse strong chromogranin A positivity (A) Immunohistochemistry demonstrating diffuse strong chromogranin A positivity within the tumor cells, highlighting the nested architecture of the lesion (X40 magnification). (B) Higher-power highlighting strong brown granular (dot-like) cytoplasmic Chromogranin A immunoreactivity, consistent with neuroendocrine differentiation (X400 magnification).

An esophagogastroduodenoscopy (OGD) revealed a medium-sized, broad-based ulcerated mass located at the gastric fundus, with a provisional diagnosis of gastrointestinal stromal tumor (GIST) (Day 2). The diagnosis was based on the formal endoscopic report, as the original endoscopic images were not retrievable at the time of manuscript revision. Endoscopic biopsy and histopathological examination demonstrated a gastric fundal neuroendocrine tumor, WHO Grade 1 (Figure 2). Mitotic activity was low (<2 mitoses per 10 high-power fields), and grading was based on mitotic count and morphology, as Ki-67 immunostaining was not available in our setting. Immunohistochemistry showed diffuse strong positivity for chromogranin A, confirming neuroendocrine differentiation (Figures 3A, 3B) [5].

Baseline laboratory investigations, including complete blood count, renal function tests, electrolytes, and tumor markers, were within normal limits (Table 1).

**Table 1:** Laboratory findings

Parameter	Patient Value	Reference Range	Interpretation
Hemoglobin (Hb)	13.0 g/dL	12.0–17.0 g/dL	Normal
White blood cells (WBC)	9.61 ×10⁹/L	4.0–11.0 ×10⁹/L	Normal
Platelets	263 ×10⁹/L	150–400 ×10⁹/L	Normal
Serum creatinine	99.7 µmol/L	64–104 µmol/L	Normal
Sodium (Na⁺)	137 mmol/L	135–145 mmol/L	Normal
Potassium (K⁺)	4.7 mmol/L	3.5–5.0 mmol/L	Normal
Carcinoembryonic antigen (CEA)	1.7 ng/mL	<5.0 ng/mL	Normal
Carbohydrate antigen 19-9 (CA 19-9)	18 U/mL	<37 U/mL	Normal

**Figure 4:**
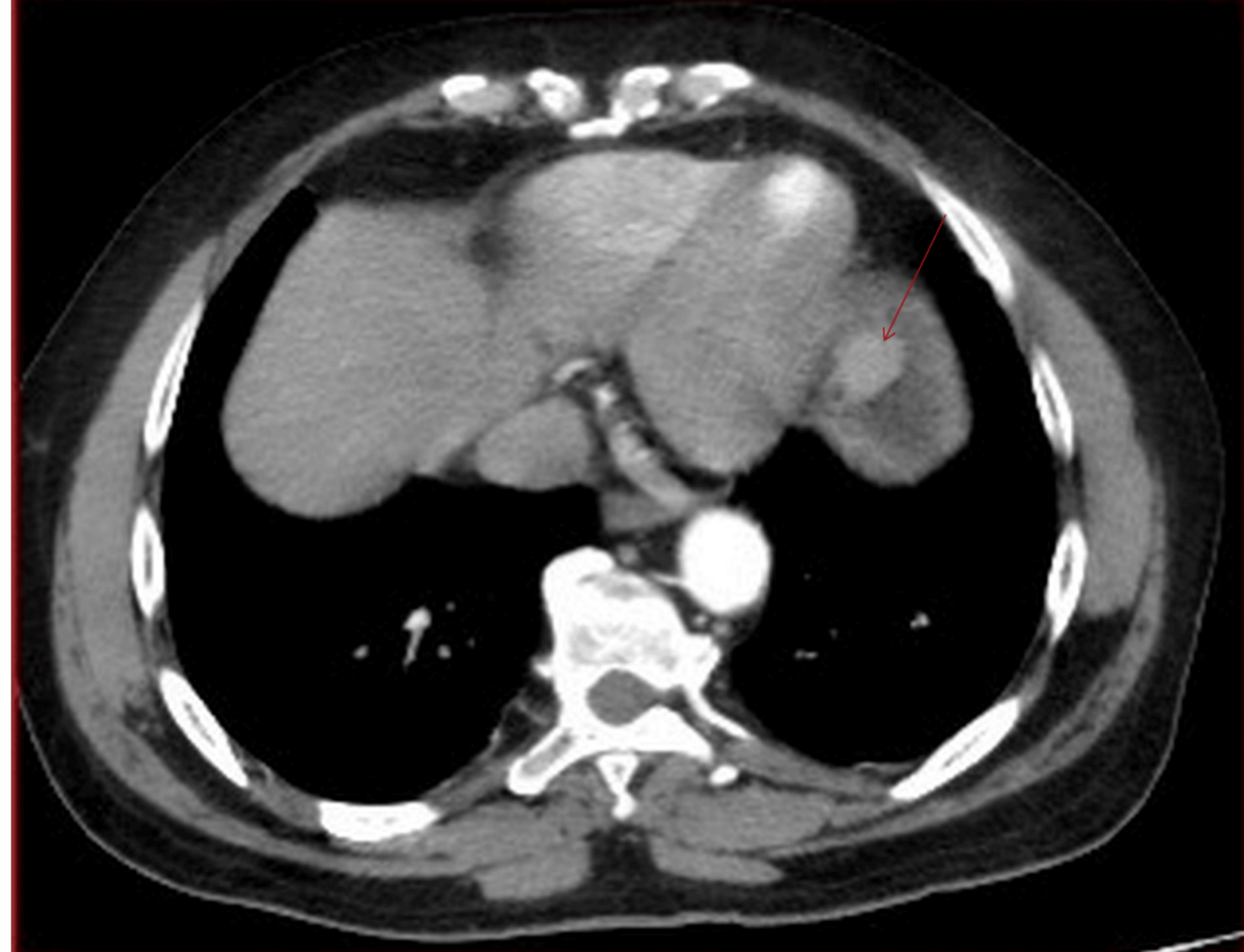
Contrast-enhanced CT scan of the chest and abdomen showing a gastric fundal mass A well-defined, rounded mass measuring 2.4 cm X 1.8 cm with homogeneous moderate enhancement is located at the gastric fundus. The mass shows an endoluminal growth pattern.

Contrast-enhanced computed tomography (CT) of the chest and abdomen demonstrated a well-distended stomach with a focal, well-circumscribed soft-tissue mass arising from the gastric fundus, measuring 2.4 cm X 1.8 cm. The radiological impression suggested a submucosal gastric fundal mass, likely GIST, with no lymph node involvement or distant metastasis (Figure 4).

A diagnosis of T2N0M0 WHO Grade 1 gastric neuroendocrine tumor was established. After counseling, the patient underwent an exploratory laparotomy with partial gastrectomy on Day 85 from initial presentation (Figure 1). The interval between diagnosis and surgery was due to preoperative optimization, completion of staging investigations, multidisciplinary evaluation, and scheduling availability.

Intra-operatively, two polypoid lesions were identified: a 1 cm sub-serosal lesion along the lesser curvature near the cardia and a 3 cm submucosal fundal lesion. Both were excised, and the stomach was closed primarily. Histopathological examination confirmed a well-differentiated gastric neuroendocrine tumor, WHO Grade 1, with clear surgical margins.

The patient was reviewed on Day 104 from initial presentation (two weeks postoperatively) and reported only mild incisional pain. He was scheduled for three-month surveillance with repeat CT scan and OGD, in accordance with recommended follow-up protocols [6].

## Discussion

Gastric neuroendocrine tumors are rare but increasingly recognized entities due to widespread endoscopic screening [1,2]. They are classified into three major subtypes based on pathogenesis and biological behavior: Type I (associated with chronic atrophic gastritis), Type II (associated with hypergastrinemia and gastrinoma), and Type III (sporadic and typically more aggressive) [3,7]. Type II gNETs are uncommon, accounting for approximately 5-6% of cases, and are frequently associated with Zollinger-Ellison syndrome and multiple endocrine neoplasia type 1 [7].

In the present case, the tumor was classified as a WHO Grade 1 well-differentiated neuroendocrine tumor based on histological characteristics and strong chromogranin A positivity [5]. Immunohistochemical staining remains essential in confirming neuroendocrine differentiation and distinguishing gNETs from other submucosal lesions [5].

Clinically, gNETs often present with non-specific symptoms, such as epigastric pain or gastrointestinal bleeding, as observed in our patient [2]. Radiologically and endoscopically, these tumors may mimic gastrointestinal stromal tumors, leading to diagnostic uncertainty prior to tissue confirmation [4]. In our case, both OGD and CT imaging suggested a GIST, highlighting the importance of biopsy and histopathological evaluation.

Tumor size, depth of invasion, and grade are critical determinants of management and prognosis. For localized lesions larger than 2 cm or those suspected of invasion, surgical resection is recommended [4,6]. Complete excision with negative margins offers excellent long-term outcomes in low-grade disease [4]. Reported five-year survival rates for localized, well-differentiated gastric neuroendocrine tumors exceed 90% following adequate surgical management [2].

This case underscores the diagnostic challenge posed by gastric submucosal masses and emphasizes the need for histopathological confirmation before definitive management. Furthermore, it contributes to the limited literature on gNETs from sub-Saharan Africa and represents, to the best of our knowledge, the first documented case reported in Tanzania.

## Conclusions

Gastric neuroendocrine tumors are rare and may present with non-specific gastrointestinal symptoms, posing diagnostic challenges. This case highlights the importance of histopathological confirmation and immunohistochemistry in differentiating gNETs from other gastric submucosal tumors. Early surgical intervention with complete resection offers favorable outcomes in patients with localized, low-grade disease.
